# Intracardiac Echocardiography: An Invaluable Tool in Electrophysiological Interventions for Atrial Fibrillation and Supraventricular Tachycardia

**DOI:** 10.31083/j.rcm2506191

**Published:** 2024-05-27

**Authors:** Tong Hu, Tongshuai Chen, Kellina Maduray, Wenqiang Han, Jingquan Zhong

**Affiliations:** ^1^National Key Laboratory for Innovation and Transformation of Luobing Theory; The Key Laboratory of Cardiovascular Remodeling and Function Research, Chinese Ministry of Education, Chinese National Health Commission and Chinese Academy of Medical Sciences; Department of Cardiology, Qilu Hospital of Shandong University, 250012 Jinan, Shandong, China; ^2^Department of Cardiology, Qilu Hospital (Qingdao), Cheeloo College of Medicine, Shandong University, 266035 Qingdao, Shandong, China

**Keywords:** intracardiac echocardiography, electrophysiological intervention, atrial fibrillation, supraventricular tachycardias

## Abstract

Researchers have investigated ways to develop optimal imaging techniques to increase the safety and effectiveness of electrophysiological (EP) procedures. Intracardiac echocardiography (ICE) is an advanced imaging tool that can directly visualize cardiac anatomical structures in high resolution, assess tissue heterogeneity and arrhythmogenic substrates, locate intracardiac catheters, monitor catheter-tissue contact and ablation injury in real-time, excluding intracardiac thrombi, and quickly detect procedural complications. Additionally, real-time imaging via ICE can be integrated with a three-dimensional (3D) electroanatomical mapping (EAM) system to reconstruct cardiac anatomy. This technique also promotes the development of zero-radiation EP procedures. Many EP studies and procedures have implemented ICE because it has several advantages over fluoroscopy and transesophageal echocardiography (TEE). ICE-guided EP procedures can be performed under conscious sedation; esophageal intubation and additional anesthesiologists are not required. Atrial fibrillation (AF) and supraventricular tachycardias (SVT) are the most common tachyarrhythmias in clinical settings. A comprehensive understanding of critical anatomical structures, such as the atrial septum, fossa ovalis (FO), and great heart vessels, is needed for the successful catheter ablation of these arrhythmias.

## 1. Introduction

Intracardiac echography (ICE) is a visual imaging tool widely applied in interventional cardiology since its introduction half a century ago. Real-time ICE is a transformative technology that can visualize and obtain high-resolution images of cardiac structures [1].

The most commonly used imaging technology in electrophysiological (EP) laboratories is fluoroscopy; however, fluoroscopy cannot visualize cardiac structures clearly. Transesophageal echocardiography (TEE) allows clinicians to capture detailed images of anatomical landmarks; however, the technique requires the patients to undergo general anesthesia and may cause mechanical trauma to the esophagus. ICE possesses several advantages over existing imaging modalities. In the USA, ICE has become a standard imaging modality in EP laboratories [2].

Atrial fibrillation (AF) is the most commonly occurring sustained cardiac arrhythmia and significantly increases the risk of death, stroke, heart failure, cognitive dysfunction, and dementia. The prevalence of AF increases with age; it is around 5.4% in males and 4.9% in females above 75 years old [3]. Supraventricular tachycardia (SVT) is used to describe various tachycardias (atrial tachycardia, atrial flutter, and paroxysmal SVT), except ventricular tachycardia and AF [4]. Catheter ablation is the first-line treatment for drug-refractory SVT and is strongly recommended in clinical practice guidelines owing to its effectiveness in treating AF.

This review article discusses the applications of ICE in EP procedures for AF and SVT.

## 2. Types of ICE Systems and How They Work

Rotating ICE catheters were designed and clinically utilized for the first time in the 1980s [5]. These devices allow high-resolution imaging of cardiac structures. However, the probe has a high frequency and poor tissue penetration ability, meaning ideal images of cardiac anatomy cannot be obtained.

The most widely used ICE system in EP labs is phased-array ICE, where a 64-element transducer is inserted in 8 Fr or 10 Fr catheters to provide a 90° sector view. The steerable catheter can be deflected in four directions (right, left, anterior, and posterior). The phased-array ICE system has several advantages over rotating ICE, including greater penetration depth, variable frequency, doppler capability (color flow, pulse, and continuous wave), and better manipulability.

**Fig. 1.**
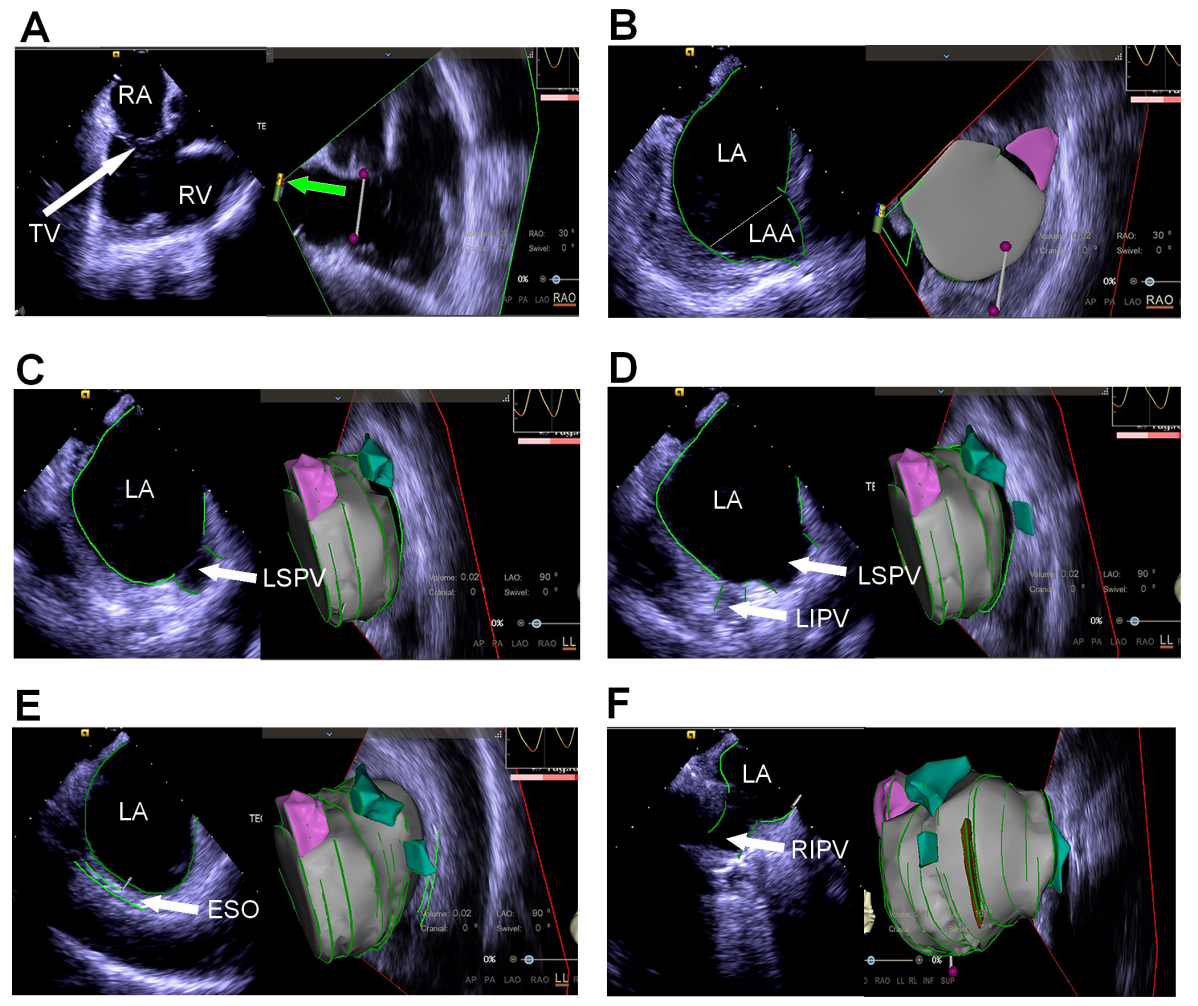
**Reconstruction of the left atrium under intracardiac echocardiography (ICE) guidance. **(A) The “home view” is obtained with the ICE probe placed in the mid-right atrium and the transducer (green arrow) facing the tricuspid valve annulus. (B) Reconstruction of the left atrium and left atrial appendage (the left part of the figure shows the image sector, with the three-dimensional reconstruction on the right). (C) Reconstruction of the left atrium and left superior pulmonary vein. (D) Reconstruction of the left atrium and left superior and inferior pulmonary veins. (E) Reconstruction of the left atrium and esophagus. (F) Reconstruction of the left atrium and right inferior pulmonary vein. RA, right atrium; TV, tricuspid valve; RV, right ventricle; LA, left atrium; LAA, left atrial appendage; LSPV, left superior pulmonary vein; LIPV, left inferior pulmonary vein; ESO, esophagus; RIPV, right inferior pulmonary vein; RAO, right anterior oblique; LAO, left anterior oblique; LL, left lateral; INF, inferior; SUP, superior; AP, Anterior-Posterior; PA, Posterior-Anterior.

ICE allows for non-contact three-dimensional (3D) reconstruction without limitations imposed by the catheter’s position. Thus, the cardiac structure can be reconstructed by rotating the catheter (Fig. 1). Some types of ICE catheters have embedded magnetic sensors, which enable the tip of the ICE catheter to be visualized on the mapping system (Fig. 1A). Anatomical landmarks can be imaged with the ultrasound catheter placed in the right atrium (RA), right ventricle (RV), or left atrium (LA). A “home view” sector is acquired with an ultrasound probe in the middle of the RA. Critical anatomical features are identified by bending the ICE catheter or rotating it from the “home view” (Fig. 1). If the anatomical features of the atrial septum or fossa ovalis (FO) are unfavorable, the ICE probe can be placed in the coronary sinus (CS). CS echocardiography facilitates excellent delineation of the anatomical features of LA, left atrial appendage (LAA), left ventricle (LV), and mitral annulus [6]. However, ICE catheters in the CS should be manipulated carefully since it has a fragile wall.

## 3. The Role of ICE in EP Procedures 

The general applications of ICE in EP procedures can be summarized as follows. (i) High-resolution visualization of anatomical landmarks; (ii) navigation and location of catheters and delivery sheaths; (iii) evaluation of catheter–tissue contact, monitoring of ablation lesion, microbubble formation, and subsequent steam pop; (iv) reduction of ionizing radiation exposure and avoidance of the need for general anesthesia; (v) rapid detection of complications.

## 4. Transseptal Puncture

In the clinical setting, transseptal puncture (TSP) is commonly performed under fluoroscopy guidance. Precise puncture is necessary to ablate left-sided arrhythmias and implant the LAA occlusion device. Visualizing the anatomy of the atrial septum and its surroundings is suboptimal using fluoroscopy. Patients and interventional electrophysiologists are exposed to radiation during the fluoroscopy-guided TSP procedure, while radiation exposure and puncture time increase during complex procedures. Since the ICE probe can directly capture images of the septum and puncture needle, determining the optimal position for TSPs is easy.

**Fig. 2.**
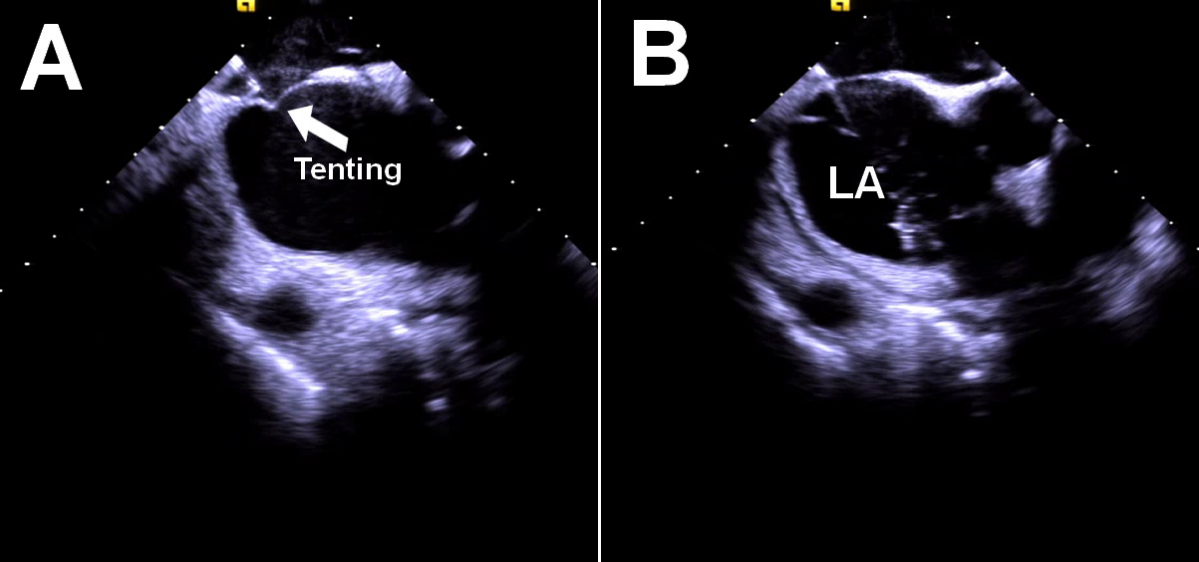
**Transseptal puncture under the guidance of ICE imaging.** (A) “Tenting” can be seen prior to transseptal puncture. (B) Saline injection confirmed that the puncture needle passed through the atrial septum into the LA. LA, left atrium; ICE, intracardiac echocardiography.

Before performing the ICE-guided TSP, the ultrasound probe is placed in the RA to exclude LA thrombus and obtain a detailed image of the FO anatomy and its surrounding structures (e.g., LA, aortic root), with a rotation of the ICE catheter. The needle–dilator–sheath assembly is placed in the superior vena cava and then pulled down to the FO after two “jumps”. When conducting real-time imaging through ICE, the tip of the puncture needle rests against the interatrial septum, shaped like a “tent” (Fig. 2A). Once the needle pass through the septum, the ‘tent’ collapses, and the needle becomes visible in the LA. To confirm the needle’s position, heparinized saline can be injected. (Fig. 2B). The injected saline bubble can be directly visualized by ICE.

It is important to rule out intracardiac thrombus before ablation of atrial arrhythmias. Anter *et al*. [7] performed a prospective blinded study to compare the diagnostic sensitivity of ICE and TEE in detecting appendage thrombi. It was observed that ICE offered comparable or better value than TEE in diagnosing intracardiac thrombi. Additionally, the ICE imaging quality of LAA was most favorable from the pulmonary artery position.

**Fig. 3.**
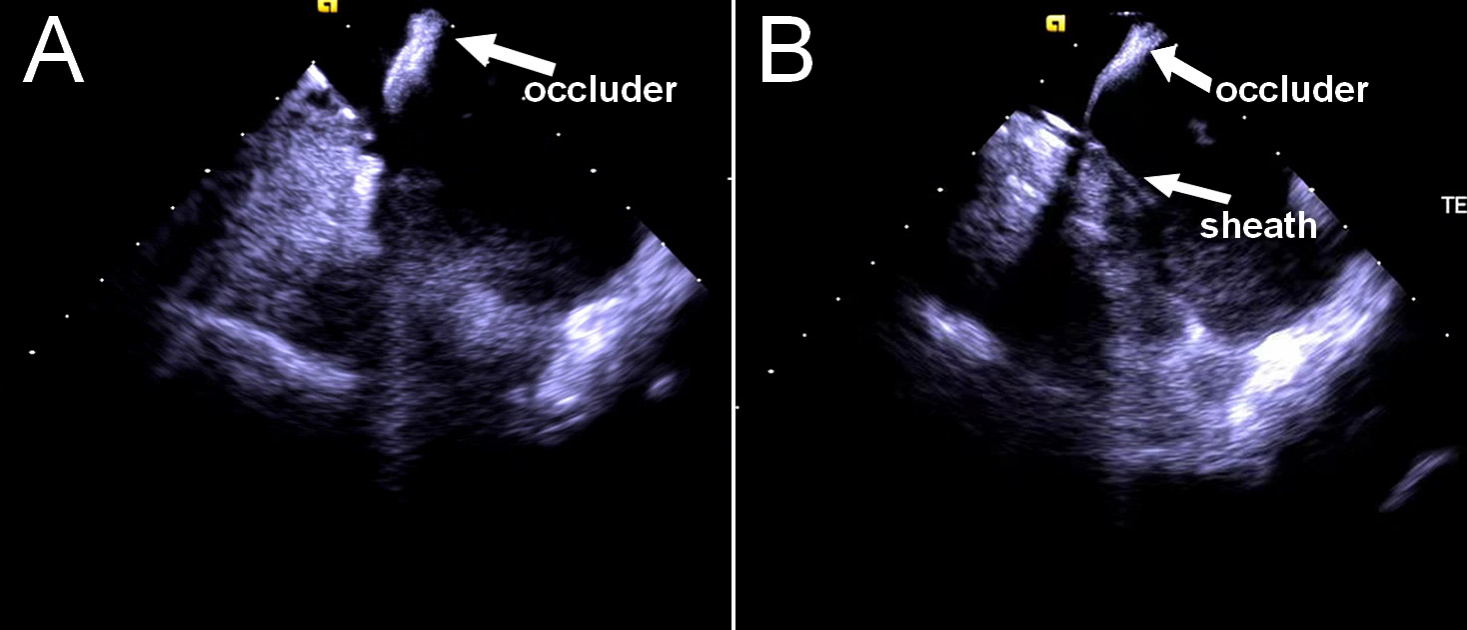
**Transseptal puncture in a patient with an atrial septal defect occluder.** The occluder (A) and needle–dilator–sheath assembly (B) can be visualized by ICE probe. ICE, intracardiac echocardiography.

Cardiac tamponade is the most common fatal complication of TSP, with an incidence of 0.09–1% [8, 9]. Other life-threatening complications include aortic root perforation, cardiac perforation, and puncture of atria [9, 10]. The risks of the aforementioned complications increase in the presence of altered septal anatomy. Phased-array ICE can be a useful tool for assissting TSP in challenging septal anatomies such as atrial septal aneurysm, the presence of an atrial septal defect occluder (Fig. 3), thick septum, enlarged atria, aortic root dilatation, and prior septal repair or puncture [8]; thus, it is an important technique to minimize complications following a puncture. Bottoni *et al*. [11] conducted a large comparative study and analyzed 2181 TSPs performed on 1862 patients. A significant difference was found between the complication rates related to TSP of groups undergoing TSP with and without ICE (0.9% vs. 3.1%, *p* < 0.001). They also found that the use of ICE was an independent predictor of TSP complications (Odds ratio (OR): 0.24, 95% CI: 0.11–0.49; *p* < 0.001).

Although the ICE-guided TSP technique has been applied to patients with cardiac implantable electronic devices (CIEDs), the outcomes are limited [12, 13, 14]. Given the risk of lead dislocation, the electrophysiologist should be careful when manipulating the mapping or ablation catheters in this specific patient population.

## 5. Atrial Fibrillation

Phased-array ICE has multiple unique practical applications in AF ablation, including a detailed assessment of pulmonary vein (PV) anatomy, monitoring PV blood flow, and avoiding PV stenosis [2, 15, 16]. Using ICE during AF ablation can decrease complication rates and hospital stays, although it considerably increases healthcare costs [17, 18]. In another study, patients who benefited from ICE experienced a 12% decrease in 90-day readmissions [19]. The higher healthcare costs associated with ICE may be partly mitigated by the extended duration of hospitalization observed in the non-ICE group [19].

Some studies have shown that ICE-guided AF ablation is associated with significantly lower X-ray exposure without reducing the effectiveness of the operation compared to the traditional approach [20, 21]. A study from Romania reported that the procedural use of ICE could decrease the fluoroscopy dose during AF ablation from the beginning of the learning curve [22]. A gradual decrease in radiation exposure dose was observed along the learning curve [22, 23]. Several studies have shown that using ICE does not prolong the operation time of AF ablation [24, 25]. Several studies have reported comparable outcomes regarding AF recurrence, whether or not ICE is used [25, 26, 27]. Pimentel *et al*. [18] found that using ICE was associated with a significantly lower incidence of repeat procedure one year after AF ablation (7.4% vs. 11.5%, hazard ratio (HR) = 0.64, 95% CI: 0.49–0.83, *p* < 0.001). A similar finding was reported in another study, which used data derived from Medicare fee-for-service claims (5.7% vs. 8.5%, adjusted HR= 0.59, 95% CI: 0.37–0.92, *p* = 0.02) [28].

**Fig. 4.**
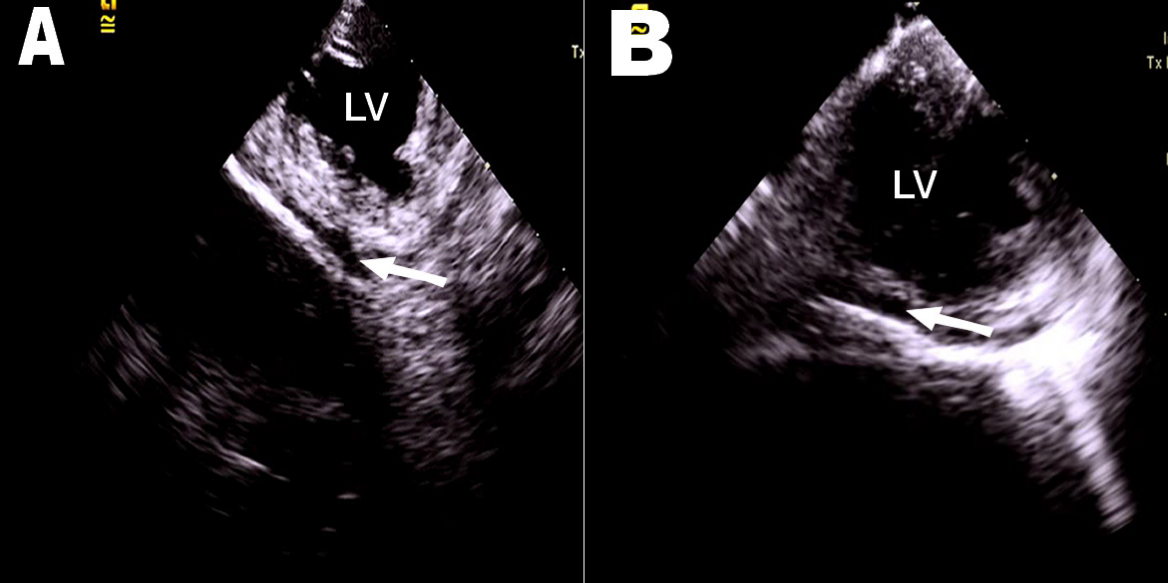
**ICE probe placed in mitral annulus.** (A) and (B) show that small amounts of pericardial effusion were detected by ICE (white arrowed). LV, left ventricle; ICE, intracardiac echocardiography.

Right phrenic nerve injury, occurring in 5% of patients undergoing superior vena cava isolation, has prompted significant efforts toward its timely detection during AF ablation [29]. In a study by Liu *et al*. [29], real-time ICE imaging provided adequate visualization of the right phrenic nerve in 35 (92%) patients and none of the 35 patients developed right phrenic nerve injury. ICE imaging permits the immediate diagnosis of pericardial effusion (Fig. 4) and can direct urgent pericardiocentesis to prevent the development of cardiac tamponade. Patients who did not undergo AF ablation with the assistance of ICE had a nearly five-fold higher risk of cardiac perforation compared to those who underwent AF ablation under the guidance of ICE [30].

Cryoballoon (CB) ablation is not inferior to radiofrequency catheter ablation (RFCA) in achieving pulmonary vein isolation (PVI) and has the advantage of shorter procedure durations compared to RFCA [31, 32, 33, 34, 35]. A single-center retrospective cohort study found that the overall dose-area product of the CB ablation procedure could be decreased by more than 95% by combining ICE imaging with an optimized fluoroscopy protocol, including omitting preprocedural imaging frame rates and removing the grid from the X-ray detector [36].

## 6. Atrial Tachycardia

Focal atrial tachycardia (AT) constitutes approximately 10% of SVT [37]. Focal ATs have a specific anatomical distribution rather than occurring randomly throughout the atria. Focal ATs occur mostly at the crista terminalis, mitral annulus, tricuspid annulus, CS ostium, and interatrial septum [38, 39].

Focal ATs originating from the atrial appendages accounted for approximately 7% of all ATs [40, 41]. Eliminating focal ATs originating from the atrial appendages, especially in the distal portion, is difficult. Most patients with these foci need re-ablation or atrial appendectomy. The unique anatomical configuration of atrial appendages hinders consistent catheter–tissue contact. A study found that the recurrence rate of AT catheter ablation in the atrial appendage can be up to 20% [42]. Real-time ICE can be used to visualize the structures of the atrial appendages at high resolution, realize noncontact anatomical reconstruction, especially in the auricle lobular local region, to ensure stable catheter tip–tissue contact, and, therefore, monitor the formation of effective ablation injuries. Additionally, intracardiac ultrasound can help accurately visualize the coronary artery adjacent to the atrial appendage, thus, increasing the safety of the ablation procedure [43]. In a pilot study, 20 patients with focal ATs originating from the atrial appendages were included [44]. Anatomical reconstruction and activation sequence mapping of the atrial appendage, guided by ICE, were performed in all patients. The contact force catheters were used for ablation. The average catheter pressure under the guidance of ICE was 7.25 ± 1.33 g, avoiding mechanical termination of AT. Recurrence occurred in three patients during a follow-up period of six months.

AT rarely originates near the atrioventricular node [45]. In cases where it does, ablation is a significant challenge. Mlčochová *et al*. [46] reported two patients with focal AT originating near the atrioventricular node; catheter ablation from the non-coronary cusp was successfully performed in both patients. The findings of that study showed that combining phased-array ICE with a mapping system can help guide the ablation catheter and assess the anatomical relationship between the aorta and its surrounding structures.

## 7. Atrial Flutter

Atrial flutter (AFL) can be classified into two types: Typical or isthmus-dependent AFL and atypical AFL. The complex cavotricuspid isthmus (CTI) encompasses the region extending from the tricuspid annulus to the orifice of the inferior vena cava. Its slow conduction serves as the EP mechanism underlying typical AFL.

The ablation of typical AFL is performed mainly to produce a bidirectional block in the tricuspid isthmus. The tricuspid annulus is difficult to visualize under fluoroscopy. The ICE probe can be used to directly visualize the specific anatomical CTI structures in real-time, assist the procedure, and improve the procedural success rate. Herman *et al*. [47] discovered that using ICE during AFL ablation was associated with decreased radiation exposure, although it did not shorten the radiofrequency energy delivery time or the overall procedure time. Another randomized trial showed that ICE-guided CTI ablation was associated with shorter procedure time and radiofrequency ablation time, as well as lower exposure to radiation [48]. Similarly, a study reported that ICE-guided ablation of the CTI in patients with typical AFL decreased the procedure time and reduced exposure to fluoroscopy [49].

In some patients, a prominent or deep pouch is present between the tricuspid annulus and the Eustachian ridge, where the ablation catheter is difficult to place. Although the myocardium of the sub-Eustachian pouch is not thick, temperature and impedance increase rapidly because of poor blood flow in the pouch and unfavorable energy delivery, resulting in incomplete ablation [50]. These reasons lead to a gap in the CTI ablation line, preventing a complete bidirectional conduction block. Hisazaki *et al*. [51] suggested that ICE was superior to right atrial angiography in evaluating the anatomical CTI structure, especially the pouch and the ridge.

The right coronary artery (RCA) usually travels within the region of the CTI [52]. An injury to the RCA induced by ablation may lead to inferior ST-elevation myocardial infarction [43]. The image of the RCA can be visualized by manually rotating the ultrasound probe clockwise or counterclockwise in the dimension of the CTI, thus, decreasing the risk of damage.

Due to a high risk of TSP, poor catheter stability, and complex EP mechanisms, catheter ablation is difficult in patients with AFL after cardiothoracic surgery. Following the surgery, a scar may develop on the atrial septum, which might alter the structure of the FO. In such cases, fluoroscopy cannot accurately localize the FO. Instead, ICE can be used to visualize the atrial septum and surrounding structures, which help accurately localize the puncture site.

## 8. Left Atrial Appendage Closure

Previous studies have suggested that the LAA is the primary source of thrombus in patients with non-valvular AF [53]. Pivotal trials have confirmed that LAA closure (LAAC) has a non-inferior efficacy in preventing stroke compared to oral anticoagulation treatment in patients with poor medication compliance or contraindications to anticoagulation [54, 55, 56]. The LAAC procedure should be performed with an imaging-guided approach [57]. TEE is an intraprocedural imaging tool with four standard scanning views (0°, 45°, 90°, and 135°) and has been implemented for a long time. However, interest in ICE-guided transcatheter LAAC procedures has increased over the past decade. Real-time ICE imaging can be performed to rule out thrombus, monitor intraprocedural complications, guide transseptal puncture, and assess the immediate effectiveness of LAA occlusion.

Some EP centers have performed the combined procedure of AF ablation and LAAC, aiming to restore sinus rhythm and prevent ischemic stroke using a single procedure. In 2020, Phillips *et al*. [58] published the long-term (726 ± 91 days) follow-up results of a multicenter study involving 142 patients who underwent the combined AF ablation and LAAC procedure. The study achieved a successful LAAC rate of 99.3% among the patients. The 30-day device and/or procedure-related major adverse event rate was 2.1%. At the last follow-up, 92% of the patients did not receive oral anticoagulation. Fassini *et al*. [59] conducted a single-center study to investigate the long-term outcome of combining cryoballoon ablation and LAAC. The study enrolled 49 patients who all achieved successful PVI and LAAC without any major procedural complications. After 24 months of follow-up, 60% of the patients did not experience atrial arrhythmia. The annualized rate of stroke and bleeding was 1% and 2%, respectively. Some studies have demonstrated that ICE-guided procedures that combined AF ablation and LAAC were safe and feasible [60, 61, 62]. In the study by Chen *et al*. [60], 56 AF patients underwent combined radiofrequency ablation and LAAC. Success rates of catheter ablation and LAAC were 100%, while the procedural adverse event rate was 3.6%. At the 12-month follow-up, 75.0% of patients remained free from atrial arrhythmia, while 96.4% did not receive oral anticoagulation [60].

Unlike TEE, ICE-guided LAA occlusion can be performed under conscious sedation, and the ICE catheter can be manipulated by a single cardiologist. Thus, a dedicated anesthesiologist or ultrasound physician is not required. Several studies have shown that LAAC can be effectively and safely guided by ICE [63, 64, 65]. By comparing ICE and TEE during the percutaneous LAAC procedure, several meta-analysis studies have suggested that ICE can provide a favorable efficacy/safety profile [66, 67, 68].

**Fig. 5.**
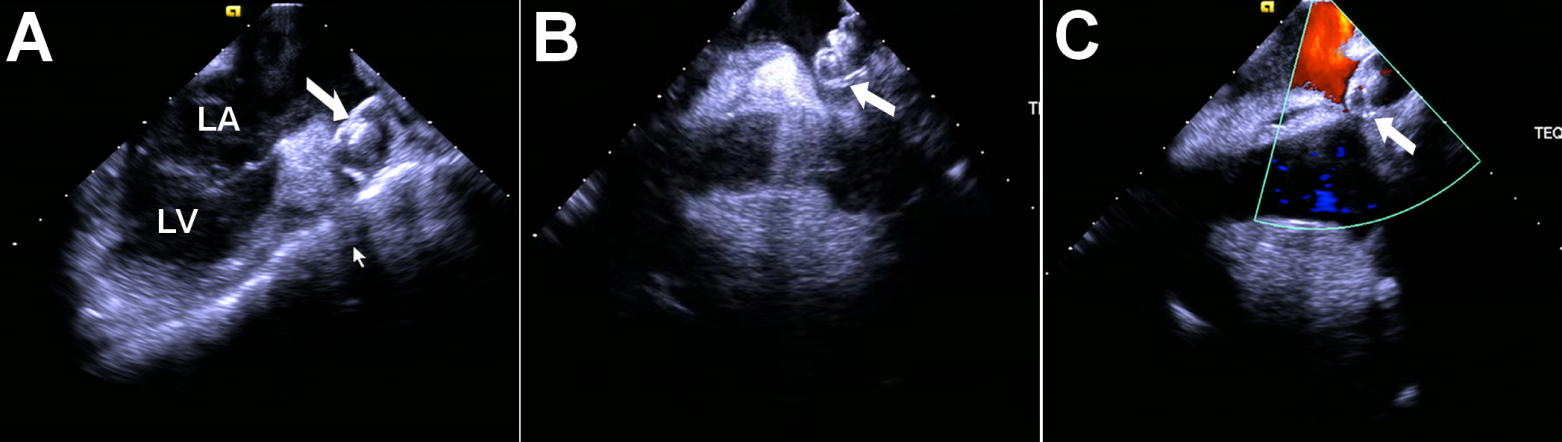
**ICE-guided LAAC procedure.** ICE probe was positioned in LA (A) and mitral annulus (B) to assess the position of the LAA occluder. (C) Peri-device leakage was evaluated by color Doppler ICE imaging. LA, left atrium; LV, left ventricle; ICE, intracardiac echocardiography; LAA, left atrial appendage; LAAC, LAA closure.

The general procedures of ICE-guided LAAC are as follows [15, 57]. (i) The ICE probe is placed in the RA, and a 3D anatomical structure of the LA is acquired. (ii) The probe is then moved to the LA through a single or double transseptal approach. (iii) ICE is used to visualize and measure the volume, morphology, ostium diameter, and depth of the LAA. (iv) The LAA occlusion device is deployed under the real-time monitoring of ICE (Fig. 5). The position, compression, and stability of the device are assessed. Color Doppler echocardiography is performed to detect the presence of peri-device leakage (Fig. 5).

Several reasons have restricted the clinical generalization of ICE-guided LAA occlusion. First, most of the available ICE catheters only have two-dimensional (2D) ultrasound imaging capabilities, and the imaging quality of LAA is generally inferior to that of TEE. An ICE probe should be placed in the retroflex LA, supramitral, and left upper PV to perform simulations of standard TEE images [57]. In the ICE LAA study, Nielsen-Kudsk *et al*. [64] simplified the imaging technique of ICE in guiding LAA closure. They scanned the LAA in a mid-LA and a supramitral view using ICE probes. In total, 100 patients received transcatheter LAA closure. The device was successfully implanted in all patients without severe intra-operative complications, and peri-device leak >5 mm was not detected at the 45-day follow-up [64]. The ICE–guided LAAC approach takes time to learn, and previous studies that reported satisfactory outcomes often involved experienced operators who frequently performed these procedures. The placement of ICE probes further complicates the operation, and a consensus on the optimal views of the LAA, standard imaging protocols, and device assessment is lacking.

## 9. Paroxysmal Supraventricular Tachycardia

Paroxysmal SVT refers to atrioventricular nodal re-entry tachycardia (AVNRT) and accessory pathway (AP)-mediated tachycardia. Catheter ablation is safe and effective for treating AVNRT. High-resolution phased-array ICE is mainly applied to reduce the exposure to ionized radiation in this setting. Luani *et al*. [69] reported a technique for slow pathway ablation in AVNRT under the guidance of ICE. ICE facilitated the direct visualization of anatomical structures within Koch’s triangle. Although the catheter placement time was significantly longer in the ICE group (2.2 ± 1.6 min vs. 12.0 ± 7.5 min, *p* < 0.05), the cryo-application duration was shorter in the ICE group (27.5 ± 37.0 min vs. 38.1 ± 33.9 min, *p* < 0.05). Moreover, acute and long-term procedural success did not differ significantly between the groups. In contrast, a randomized study suggested that using ICE was associated with a significantly shorter time for mapping and ablation [70]. A single-center randomized trial by Bocz *et al*. [71] compared ICE-guided vs. EAM-guided slow pathway ablation. Their study demonstrated that ICE guidance during slow pathway ablation resulted in significantly shorter procedural times and radiofrequency delivery than the electroanatomical mapping (EAM)-guided system [71]. They also emphasized that Koch’s triangle can be directly visualized by ICE, especially in challenging cases.

Although catheter ablation of AP is a common and routine EP procedure, performing it using traditional imaging modalities might be difficult in some cases. AP ablation under ICE and 3D mapping guidance was first performed on a male with prior procedural failure [72]. Treating typical right free-wall AP presents a complex challenge due to lower ablation catheter stability at the tricuspid annulus and a success rate lower than that recorded for left-sided or septal AP [73]. To address this problem, Jan *et al*. [73] developed a “loop” maneuver with the integrated use of ICE and EAM. Their findings suggested that this novel technique may facilitate the stability of the ablation catheter at the tricuspid annulus and provide favorable procedural outcomes.

## 10. Parahisian Arrhythmias

The parahisian (PH) region consists of several anatomical structures, including the atrioventricular node and the bundle of His, which are associated with a high risk of atrioventricular block in EP procedures [74]. The PH region is bounded by the aorta, tricuspid, and mitral annulus structures alongside the bundle of His and ventricular outflow tracts [75]. Supraventricular arrhythmias from the PH region are mainly AP and AT.

Real-time ICE visualization provides a comprehensive understanding of the complex anatomy of PH, thus, increasing the safety of ablation in this area. ICE imaging of the PH region can be performed at four positions: Mid-RA, low lateral RA, CS, and RV inflow [74]. The ICE probe placed in the middle of the RA provides a view of the long axis of the aorta and the septal aspect of the RV, whereas the ICE probe located in the RV inflow captures the cross-section of the aorta [74]. Using ICE imaging obtained from the low lateral RA and CS, the interventricular septum and the PH region can be visualized [74]. A single-center study analyzed 34 patients undergoing ablation for parahisian AT from RA, LA, or non-coronary cusp (NCC) with the support of ICE imaging [76]. Acute successful ablation was achieved in 33 cases. Atrioventricular block occurred in two patients after RA ablation, with no complications occurring in the NCC and LA approaches. ICE demonstrated that the ablation catheter was more stable in the NCC approach.

## 11. Arrhythmia in Patients with Congenital Heart Disease

Patients with postoperative congenital heart disease (CHD) are prone to arrhythmia. CHD affects around one million adults in the United States [77]; surgical scarring and abnormal anatomy predispose these patients to arrhythmia. Several congenital heart defects are susceptible to arrhythmia, including Ebstein anomaly, transposition of the great arteries, tricuspid atresia, pulmonary atresia, and tetralogy of Fallot (TOF) [78]. Mapping of repaired CHD is a complex and challenging task for electrophysiologists. Real-time ICE has unique benefits for the ablation of arrhythmias after surgical correction. The ligating sutures, baffles, and tunnels can be visualized via ICE.

AP-mediated reentrant tachycardia is prevalent in patients with Ebstein anomaly, with an incidence of 30–40% [79]. The success rate of AP ablation in patients with Ebstein anomaly is still low. However, the combined use of ICE imaging and EAM contributes to mapping AP and facilitates satisfactory ablation outcomes for this population [79, 80].

In cases of corrected transposition of the great arteries, AT is relatively uncommon [81]. In such situations, catheter ablation is complex and technically challenging owing to the reversed anatomical structures. Atrial switch surgery is the main surgical treatment implemented for the transposition of the great arteries. We reported a successful ablation of AT at the pulmonary outflow tract in a patient with congenitally corrected transposition of the great arteries (IDD type) using integrated ICE and EAM [82]. In that patient, the bilateral atria and their adjacent structures were directly reconstructed. CTI-dependent AFL is a common reentrant tachycardia that occurs after the atrial switch procedure. In cases of repaired transposition of the great arteries, the CTI is divided by an intra-atrial baffle, which results in the formation of two isthmuses: One situated between the tricuspid annulus and the baffle and the other located between the baffle and the anatomical boundary of the inferior vena cava [83]. Trans-baffle puncture may be required in some patients for CTI ablation [83]. Baffle leak after atrial switch procedures is uncommon after atrial switch procedures and may provide access for catheters [84]. The baffle leak can be clearly visualised with ICE imaging [84].

## 12. Fluoroless or Near Fluoroless EP Procedures

Long-term, low-dose radiation can promote tumorigenesis. “As low as reasonably achievable” (ALARA) is a key principle related to reducing the cumulative harm of ionizing radiation to medical staff [85]. Interventional cardiologists have a three-fold greater lifetime risk of cancer compared to the general population [86]. Meanwhile, the heavy lead apparel may increase susceptibility to orthopedic injuries. Substantial efforts have been made to reduce fluoroscopic dose in EP procedures.

Zero-fluoroscopy ablation has gained much attention and can be particularly effective for treating pregnant patients, the pediatric population, and certain patients at high risk of contrast-induced nephropathy. Zero or near-zero fluoroscopy electrophysiology procedures become a reality owing to technological advances in mapping systems, catheters, and imaging techniques. ICE is widely used by electrophysicists as a critical tool to reduce fluoroscopy. Several studies have reported that fluoroless TSP can be performed safely and effectively under the guidance of ICE imaging, with or without the integration of 3D EAM systems [12, 13, 87].

ICE-guided-reduced or zero-fluoroscopy procedures for AF are conducted in many EP centers. In 2009, Ferguson *et al*. [88] reported RFCA for AF using ICE and a 3D EAM mapping system without fluoroscopy. In their cohort, 19 patients underwent zero-fluoroscopy procedures, and fluoroscopy was used to assist TSP in the remaining two patients. Other studies also showed that ICE-guided non-fluoroscopic catheter ablation for AF is safe and feasible [25, 27, 87, 89]. A meta-analysis suggested that zero fluoroscopy catheter ablation for AF is related to significantly reduced procedure time, fluoroscopy time, and fluoroscopy exposure compared to conventional strategies [90]. Some studies reported that the learning curve for ICE-guided zero-fluoroscopic AF ablation is approximately 10–30 cases [91, 92]. The transition to the fluoroless strategy may increase the duration of the procedure; however, the procedure may take less time if the operators are experienced [89].

Ahn *et al*. [93] conducted a randomized controlled study to compare the efficacy and risk profile of zero-fluoroscopy and traditional CB ablation for paroxysmal AF. They observed a fluoroscopic time of 0.008 min and a radiation exposure dose of 29.4 cGy⋅${\mathrm{cm}}^{2}$ in patients who underwent CB ablation under the guidance of ICE. Fluoroscopy was used in only one patient in the non-fluoroscopic group; successful PVI was achieved in all subjects. Freedom from cardiac arrhythmias was similar in the fluoroless and conventional groups. The following echographic findings indicated favorable PV occlusion: (i) ICE imaging showed abundant blood bubbles from the occluded PV after the deflation of CB, and (ii) color Doppler flow imaging could not detect blood signals around the inflated balloon.

Two studies have investigated the feasibility and safety of zero-fluoroscopy CTI ablation under the guidance of ICE alone [94, 95]. Luani *et al*. [95] reported that CTI ablation guided only by ICE imaging was feasible and safe, based on a single-center study. Debreceni *et al*. [94] conducted a comparative study and reported a significant reduction in CTI ablation time under the sole guidance of ICE. The procedure time, puncture-to-first-ablation time, and total ablation energy in the ICE-guided group were similar to those in the fluoroscopy + ICE-guided group [94].

## 13. Limitations and Advances of ICE Technology 

Most images from commercially available ICE systems are limited to 2D. Some studies have shown that real-time 3D volumetric ICE (4D ICE) catheters are feasible and safe and, thus, can be used to assist TSP, PVI, and LAAC. The 4D ICE technology has multiplanar imaging capability, allowing better and more detailed visualization of anatomical landmarks [96]. The main disadvantage of the first-generation 4D ICE technology is that the ultrasound field of view is small and limited. The new generation of 4D volume ICE provides a larger imaging volume of 90° × 50° [97]. Biosense Webster, Inc. (Diamond Bar, CA, USA) has launched an ICE probe (NUVISION NAV); it is the only ICE probe that has 4D functionality and is compatible with the CARTO 3 system (CARTO, Biosense Webster Inc., Diamond Bar, CA, USA).

ICE imaging depends on the manual annotation of the ultrasound frame contour by experienced technicians. The Cartosoundfam™ (Biosense Webster Inc., Diamond Bar, CA, USA) is a novel ICE-based algorithm that can reconstruct the 3D anatomical structure of the LA from a series of 2D ICE frames obtained from the RA and right ventricular outflow tract. Moreover, it does not require a technician to annotate ultrasound contours manually [98]. A feasibility study showed that this automated algorithm can detect the LA anatomy with satisfactory accuracy [98].

The cost of the ICE probe is a major concern in less-developed regions. However, according to Hemam *et al*. [99] and Alkhouli *et al*. [63], the cost of ICE can be offset by eliminating the need for general anesthesia and TEE. Additionally, compared to the extra costs related to using EAM systems, the expenditure associated with ICE is reported to be similar [100]. Indeed, several countries permit the re-sterilization and reprocessing of ICE catheters [101]. Velagic *et al*. [102] investigated the feasibility and safety of using reprocessed ICE catheters in EP procedures (one ICE probe could be used for 19.8 EP procedures). They found that reprocessing ICE catheters can lead to cost and waste reductions of 90% and 95%, respectively, without increasing the risk of complications, such as infection and allergic reactions.

## 14. Conclusions

Innovative ICE imaging has improved the safety and efficacy of practicing EP. Advances in technology, the development of the standard view of the ICE image plane and the formulation of expert consensus could further expand the clinical application of ICE. However, different imaging modalities have unique advantages and disadvantages, meaning ICE can be combined with conventional visualization technologies to optimize procedural outcomes. The optimal imaging protocol should be individualized for each patient.
